# Comparison of Systematic Ticagrelor-Based Dual Antiplatelet Therapy to Selective Triple Antithrombotic Therapy for Left Ventricle Dysfunction Following Anterior STEMI

**DOI:** 10.1038/s41598-018-28676-4

**Published:** 2018-07-09

**Authors:** Alexandra Bastiany, Alexis Matteau, Fady El-Turaby, Alexandre Angers-Goulet, Samer Mansour, Benoit Daneault, Brian J. Potter

**Affiliations:** 1 Cardiology Service, Department of Medicine, Centre Hospitalier de l’Université de Montréal (CHUM), Montréal, QC Canada; 2 Centre de Recherche du CHUM (CRCHUM), Montréal, QC Canada; 3 Cardiology Service, Department of Medicine, Centre Hospitalier de l’Université de Sherbrooke (CHUS), Sherbrooke, QC Canada

## Abstract

Antithrombotic management of STEMI patients with apical dysfunction, but without demonstrable thrombus, is controversial. Triple antithrombotic therapy (TATT, defined as the addition of oral anticoagulation to dual antiplatelet therapy, or DAPT) may be associated with increased bleeding, while DAPT alone may not adequately protect against cardio-embolic events. We undertook a dual-center study of anterior STEMI patients treated with primary PCI (pPCI) from 2013 to 2015 and presenting presumed new apical dysfunction. The *Centre hospitalier de l’Université de Montréal* (CHUM) uses a strategy of selective TATT, whereas the *Centre hospitalier universitaire de Sherbrooke* (CHUS) has favored ticagrelor-based DAPT for all patients since 2013. The primary composite outcome consisted of death, MI, stroke, revascularization, and BARC 3 to 5 bleeding up to 4-months follow-up. We identified 177 cases (69 CHUM; 108 CHUS). Baseline characteristics were similar and procedural success was high (97%). There was no difference in post-procedure LVEF (39 ± 9% vs 37 ± 9%) or the extent of apical dysfunction. The primary composite outcome occurred in 27% with the selective TATT strategy compared to 19% with ticagrelor-DAPT (p = 0.342). Thus, this retrospective dual-center analysis does not support a strategy of conventional TATT over ticagrelor-based DAPT for patients with apical dysfunction following anterior STEMI treated with pPCI. A pragmatic randomized trial is needed to provide a definitive answer to this clinical conundrum.

## Introduction

The risk of cerebral ischemic events is higher after myocardial infarction (MI). Historically, this risk has been shown to be particularly elevated following anterior ST-elevation MI (STEMI) with regional wall motion abnormality involving the left ventricular apex^1–4^. Such patients are at an increased risk of developing left ventricular thrombus (LVT), which is thought to lead to systemic embolism (SE), including stroke^5–7^. However, the most appropriate antithrombotic therapy for preventing SE in patients with apical dysfunction but no demonstrable thrombus remains controversial, particularly for patients treated with timely primary percutaneous coronary intervention (pPCI)^8^. The addition of oral anticoagulation to standard therapy for patients with apical dysfunction without LVT received a class IIb indication (LOE C) in the most recent STEMI guidelines (uncertain benefit)^9–11^. To date, there has been no adequately powered randomized controlled trial of prophylactic triple antithrombotic therapy (TATT, consisting of the addition of anticoagulation to dual antiplatelet therapy) and but a solitary single-center retrospective study addressing this question in an exclusively pPCI population^8^. Also, to the best of our knowledge, no study to date has specifically sought to compare TATT to dual antiplatelet therapy (DAPT) with newer P2Y12-inhibitors. The objective of this retrospective study was therefore to determine the effectiveness and safety of modern ticagrelor-based DAPT without oral anticoagulation compared to a conventional strategy of selective (physician discretion) TATT, herein defined as the addition of a vitamin-K antagonist to clopidogrel-based DAPT, for anterior STEMI patients treated with pPCI and presenting apical dysfunction on subsequent transthoracic echocardiography (TTE).

## Methods

The *Centre hospitalier de l’Université de Montréal* (CHUM) and the *Centre hospitalier universitaire de Sherbrooke* (CHUS) are two large-volume, academic, tertiary care centers in the province of Québec, Canada. In response to the recent availability of ticagrelor, cardiologists at the CHUS have abandoned the practice of prophylactic anticoagulation of apical dysfunction in favor of systematic ticagrelor-based DAPT since 2013 with rare exception. In contrast, no such practice shift was noted at the CHUM, where clinicians have elected to pursue traditional clopidogrel-based TATT in selected cases of apical dysfunction. The decision to add anticoagulation to DAPT at the CHUM is typically based on a combination of the extent of apical dysfunction, the left ventricular ejection fraction (LVEF), and perceived bleeding risk, but it is not standardized (physician discretion). The care objectives for STEMI patients in these two university hospitals is otherwise identical. This study was approved by the institutional review boards of each center that provided a waiver of informed consent and was conducted in accordance with the Good Clinical Practice (GCP) guidelines.

We conducted a retrospective analysis of all patients admitted or transferred to the CHUM or CHUS from April 1^st^, 2013, to March 31^st^, 2015, with a primary diagnosis of anterior STEMI treated with pPCI and presenting presumed new apical dysfunction by TTE (≥1 dysfunctional apical segment) regardless of LVEF. All patients were identified through the catheterization laboratory procedure databases at either center. Patients were excluded if no revascularization procedure was performed, if they were revascularized by means other than by pPCI, or if TTE was not performed during the index hospitalization. Additional exclusion criteria included any established indication for anticoagulation (e.g. atrial fibrillation or demonstrable LVT on TTE) or a contra-indication to oral anticoagulation or antiplatelet therapy. All anterior STEMI patients treated with pPCI without these exclusions were included in the primary cohort. A survivorship sub-cohort of the primary cohort was also defined a priori consisting of those patients surviving to hospital discharge for whom discharge treatment would be known.

The primary efficacy outcome consisted of a “net averse clinical event” (NACE) composite outcome comprised of major adverse cardiovascular and cerebrovascular events (MACCE; death, myocardial infarction, unplanned revascularization, stroke, or transient ischemic attack) and major bleeding (Bleeding Academic Research Consortium (BARC) class 3 or 5) at 4 months^12^.

Prospectively identified secondary outcomes included the individual components of the primary composite outcome, an “irreversible events” composite outcome (death, MI, stroke, or intracerebral hemorrhage), and LVT formation. Additionally, we defined an exploratory *post hoc* “treatment failure” composite outcome variable comprised of the occurrence of the MACCE, major bleeding, or LVT formation (i.e. NACE or LVT).

Descriptive statistics are presented as the number of individuals and percent proportion for categorical variables, means and standard deviation for normally distributed continuous data, and medians and interquartile ranges for non-normally distributed continuous data. Crude rates were compared using the Fisher Exact or Chi^2^ tests, as appropriate. A Student’s t-test or the Median test were used for continuous variables, as appropriate.

Our primary analysis consisted of a comparison of the two hospital-stratified treatment strategies – ticagrelor-DAPT for all (CHUS) vs. physician-discretion TATT or DAPT (CHUM) – using multivariable logistic regression with a backwards selection algorithm (p = 0.25) with a plan to use the hospital of admission as an instrumental variable provided that significant antithrombotic practice divergence could be confirmed.

Secondary analyses limited to the survivorship sub-cohort were also conducted. First, a similar multivariable instrumental variable analysis of post-discharge events limited to those patients surviving to discharge (survivorship sub-cohort) was performed using the same methods described for the primary analysis. Second, we also sought to compare TATT to ticagrelor-DAPT irrespective of the hospital of admission in a propensity-matched sample using a greedy 1:1 matching algorithm for the likelihood of receiving TATT at discharge. Necessarily, this analysis was also limited to the survivorship sub-cohort for whom discharge therapy was known. All statistical analyses were performed using SAS 9.4 (SAS Institute Inc., Cary, North Carolina) and a p < 0.05 was considered significant for all analyses without correction in keeping with an exploratory analysis.

## Results

A total of 177 patients, aged between 22 and 91 years, met the inclusion criteria of this study. Baseline clinical and procedural characteristics are presented in Table 1. Patients at the CHUM had higher rates of hypertension and diabetes and had higher CHADS_2_ scores compared to patients at the CHUS. Clinical characteristics were otherwise overall similar. Most patients in both groups were treated with drug-eluting stents. However, treatment delay was longer and more patients experienced cardiac arrest at the CHUM. Despite this, there was no difference in post-pPCI LVEF, nor in the extent of apical dysfunction in terms of the number of affected segments between the two hospital populations.

**Table 1:** Selected Baseline Characteristics.

	CHUM (Selective TATT) N = 69	CHUS (Ticagrelor-DAPT) N = 108	Unadjustedp-value
Age (yrs)	64.6 ± 13.6	62.2 ± 10.9	0.183
Men	49 (71%)	79 (73%)	0.863
Diabetes	17 (25%)	10 (9%)	0.009*
Hypertension	37 (54%)	41 (38%)	0.045*
Dyslipidemia	31 (45%)	43 (40%)	0.534
BMI (kg/m^2^)	27.1 ± 5.2	27.0 ± 4.2	0.898
eGFR (mL/min)	84.5 ± 35.2	100.7 ± 38.5	0.007*
Hemoglobin (g/L)	142.3 ± 27.2	141.1 ± 16.3	0.706
Stroke/TIA History	2 (3%)	2 (2%)	0.641
CHADS_2_ Score	1 (0–2)	0 (0–1)	0.012*
CHA_2_DS_2_-VASC Score	1 (1–3)	1 (0–2)	0.160
HASBLED Score	1 (0–2)	1 (0–1)	0.074
Prior PCI	11 (16%)	1 (1%)	0.0002*
Radial Access	57 (83%)	81 (75%)	0.268
FMC-Device Time (min)	127 (101–178)	97 (79–121)	<0.001*
FMC-Device ≤ 90 min	15 (22%)	43 (40%)	0.021*
Drug-Eluting Stent Use	45 (65%)	84 (78%)	0.083
Procedural Success	64 (93%)	108 (100%)	0.008*
Cardiac Arrest	16 (23%)	11 (10%)	0.031*
LVEF	39% ± 9%	37% ± 9%	0.892
No. Dysf. Apical Segments	4 (3–4)	4 (4–4)	0.469
1 Dysf. Segment	1 (1%)	6 (6%)	0.293^†^
2 Dysf. Segments	9 (13%)	11 (10%)	
3 Dysf. Segments	10 (15%)	9 (8%)	
4 Dysf. Segments	49 (71%)	82 (76%)	

BMI: Body mass index. eGFR: Estimate glomerular filtration rate (Cockcroft-Gault method). TIA: Transient ischemic attack. PCI: Percutaneous coronary intervention. FMC: First medical contact. LVEF: Left ventricular ejection fraction.

*p < 0.05. ^†^Result for the Chi^2^ analysis of the number of affected apical segments.

In-hospital event rates are shown in Table 2. Three CHUM patients and 7 CHUS patients died prior to discharge. Antithrombotic treatment at discharge for surviving patients is shown in Table 3. A premise for the planned analysis was a very low rate of anticoagulation at discharge among patients at the CHUS. Indeed, only 7% of CHUS patients received TATT (akin to cross-over). The vast majority of the remaining patients were treated with ticagrelor-based DAPT. In comparison, 44% of CHUM patients surviving to discharge were treated with TATT. The other 56% were nearly equally likely to receive clopidogrel-, ticagrelor-, or prasugrel-based DAPT. Antiplatelet and anticoagulant treatment patterns were statistically different between both centers. No patient in either group was discharged on non-vitamin K oral anticoagulant (NOAC) therapy.

**Table 2:** In-Hospital Clinical Events.

	CHUM (Selective TATT) N = 69	CHUS (Ticagrelor-DAPT) N = 108	Unadjusted p-value
MACCE	7 (10%)	21 (19%)	0.139
Death	3 (4%)	7 (6%)	0.742
Myocardial Infarction	1 (1%)	0 (0%)	0.390
Unplanned Revascularization	2 (3%)	15 (14%)	0.018*
TLR	0 (0%)	5 (5%)	0.158
Non-TLR	2 (3%)	10 (9%)	0.131
Stroke/TIA	1 (1%)	0 (0%)	0.390
Major Bleeding	4 (6%)	0 (0%)	0.022*
In-Hospital Transfusion	4 (6%)	1 (1%)	0.074
LVT*	0 (0%)	0 (0%)	—

MACCE: Major adverse cardiovascular and cerebrovascular event. TLR: Target lesion revascularization. LVT: Left ventricular thrombus.

*LVT was an exclusion criterion for this analysis.

**Table 3:** Antithrombotic Treatment at Discharge.

	CHUM (Selective TATT) N = 66*	CHUS (Ticagrelor-DAPT) N = 101*	Unadjusted p-value
DAPT	37 (56%)	94 (93%)	<0.0001
Clopidogrel	15 (41%)	7 (7%)	<0.0001
Ticagrelor	13 (35%)	85 (90%)	<0.0001
Prasugrel	9 (24%)	2 (2%)	0.0002
TATT	29 (44%)	7 (7%)	<0.0001
Clopidogrel	24 (83%)	6 (86%)	1.0000
Ticagrelor	1 (3%)	1 (14%)	0.3556
Prasugrel	4 (14%)	0 (0%)	0.5658
VKA	29 (100%)	7 (100%)	—
NOAC	0 (0%)	0 (0%)	

*Three CHUM patients and 7 CHUS patients died prior to discharge.

VKA: Vitamin K antagonist; NOAC: Non-vitamin K antagonist oral anticoagulant.

Clinical follow-up at 4 months was available in 97% of patients. The rate of the primary NACE composite outcome was 27% with the selective TATT strategy (CHUM) compared to 19% with the ticagrelor-DAPT strategy (CHUS; p = 0.342). Comparison of MACCE alone (21% vs 19%, p = 0.844) was also non-significant, but there was a significant excess of major bleeding with the selective TATT strategy (7% vs 0%, p = 0.019) and a trend in terms of an increase in the irreversible events composite outcome (15% vs 6%, p = 0.104; Table 4). The results of subgroup analyses based on LVEF and the extent of apical dysfunction are shown in *Supplementary Material* (Table A1). After multivariate regression with instrumental variable analysis, admission hemoglobin predicted NACE, major bleeding, and treatment failure, whereas the presence of cardiogenic shock was an independent predictor of all composite outcomes driven by prediction of ischemic outcomes. The adjusted odds ratio for NACE with a selective TATT strategy was 1.87 (CI_95_ 0.77–4.54), which was not statistically significant (p = 0.169).

**Table 4:** Selected Primary and Secondary Outcomes (Complete Cohort).

	CHUM (Selective TATT) N = 64*	CHUS (Ticagrelor-DAPT) N = 108	Unadjusted p-value
Overall Population			
NACE	17 (27%)	21 (19%)	0.342
MACCE	13 (21%)	21 (19%)	0.844
Major Bleeding	4 (7%)	0 (0%)	0.019
Irreversible Events	9 (15%)	7 (6%)	0.104
LVT	0 (0%)	1 (2%)	1.000
Treatment Failure** (NACE or LVT)	17 (27%)	22 (20%)	0.354

*Five CHUM patients without in-hospital events did not have clinical follow-up within 4 ± 1month post-MI at either the CHUM or the identified referring center. **“Treatment Failure” was a *post hoc* outcome consisting of MACCE, Major Bleeding, or LVT.

NACE: Net adverse clinical events. MACCE: Major adverse cardiovascular and cerebrovascular events. LVT: Left ventricular thrombus. LVEF: Left ventricular ejection fraction.

The results of subgroup analyses in the survivorship sub-cohort are shown in the *Supplementary Material* (Table A2). There were no major bleeding or cerebral ischemic events identified in either group following discharge. Given the lack of post-discharge clinical events ascertained in the CHUS group, it was impossible to establish univariate and multivariate predictors of post-discharge adverse events in this survivorship sub-cohort. The propensity-matched analysis (presented in detail in the *Supplementary Material*; Table A3) did not reveal any significant differences in terms of post-discharge clinical composite outcomes between TATT and DAPT patients.

The exploratory *post hoc* “treatment failure” composite outcome occurred in 17 (27%) cases at the CHUM and 22 (20%) cases at the CHUS overall (p = 0.354), with 7(12%) and 1 (2%) post-discharge events occurring among CHUM and CHUS patients (p = 0.004), respectively. The lone patient to develop documented LVT had only 2 dysfunctional segments post-pPCI and received ticagrelor-DAPT at the CHUS. Admission hemoglobin and cardiogenic shock were again both independent predictors of “treatment failure” (NACE or LVT).

## Discussion

The results of this analysis do not appear to support a strategy of physician-discretion TATT over a strategy of systematic ticagrelor-based DAPT in an all-comers pPCI population with anterior STEMI and apical dysfunction without thrombus. While there was no statistically significant difference in the NACE primary outcome, there was a signal for possible increased harm with a selective TATT strategy. Additionally, there was a small, but statistically significant, excess of major bleeding events and a weak trend in terms of the “treatment failure” composite outcome with a selective TATT strategy. Multivariable analysis showed that admission hemoglobin was predictive of bleeding events, whereas cardiogenic shock was a strong independent predictor of ischemic risk. A propensity matched analysis of the survivorship sub-cohort – akin to an on-treatment analysis – also did not reveal any significant difference between TATT and DAPT therapy.

Prophylactic anticoagulation of anterior STEMI patients with apical dysfunction is controversial^9,10^, particularly for patients treated with pPCI and candidates for novel antiplatelet therapy^8,13,14^. In fact, the present study is, to the best of our knowledge, only the second study to address an exclusively pPCI population^8^ and the first to use a ticagrelor-based DAPT strategy as a comparator.

Despite early reports of the benefits of adding anticoagulation to standard therapy for patients with anterior MI^15–17^, no study that has included PCI patients has demonstrated an advantage of TATT^8,18–22^. Moreover, despite a recent report that the incidence of LVT following pPCI is not negligible^23^, Le May *et al*.’s propensity-matched retrospective single-center analysis suggested a higher incidence of NACE with TATT compared with clopidogrel-based DAPT^8,24^. Recently, in a mixed pPCI and pharmacoinvasive population, Shavadia *et al*. showed no significant difference in terms of MACCE or bleeding, but a somewhat unexpected benefit in terms of mortality with TATT^22^. Clinicians are therefore faced with an important clinical conundrum with little direction from the guidelines^10,25^. Recently, however, it has been suggested by some that so-called “modern” DAPT consisting of aspirin and a novel P2Y12-inhbitor, such as ticagrelor, might be a reasonable alternative to TATT in pPCI patients. The practice shift at the CHUS since 2013 is an example of the attractiveness of such a simple therapeutic solution.

The present study was therefore designed to compare two broadly applied alternative treatment strategies; one of ticagrelor-based DAPT for all anterior STEMI patients with apical dysfunction treated by pPCI and the other representing a traditional strategy of selective-TATT at the treating physician’s discretion. The total cohort analysis did not reveal a significant difference between the two strategies in terms of the primary or any secondary composite outcomes. There was, however, a significant excess of major bleeding with a selective TATT strategy that is consistent with the recent findings of Le May *et al*. and Shavadia *et al*.^8,22^.

Whether to include in-hospital events in the primary analysis may be cause for some debate. Indeed, most of the retrospective registry analyses, particularly those relying on propensity matching, needed to know treatment at discharge to properly classify patients. However, this methodological necessity may have led to the exclusion (e.g. bleeding-related death prior to discharge) or misclassification (e.g. non-mortal bleeding in a TATT patient leading to DAPT treatment at discharge) of some individuals. As a patient’s treatment at discharge is typically known prior to discharge and, in the case of both DAPT and TATT, often by the second day of admission, we believe that any in-hospital event occurring after a diagnosis of apical dysfunction may be attributed to the treatment assignment and that exclusion of these events may lead to bias. Fortunately, because we set out in our primary analysis to evaluate two treatment strategies (selective TATT vs ticagrelor-DAPT) that stratified according to the pPCI center of admission – and not treatment *per se –* patient classification was possible from the moment of study eligibility, allowing inclusion of in-hospital events in the outcome analyses. Analyses of post-discharge events in the survivorship sub-cohort, including the propensity-matched analysis, were performed in order to enhance comparability with prior studies.

Although it stands to reason that a clinically enriched population with high risk features (eg, lower LVEF and more extensive apical dysfunction) may derive more benefit from more aggressive antithrombotic therapy, we elected to use broad apical dysfunction criteria (≥1 segment) without specifying LVEF for study entry in order to evaluate the effectiveness of two treatment strategies as they are currently applied in clinical practice. In the CHUM patients, the presence of an LVEF less than 40% and at least 2 dysfunctional apical segments appeared generally sufficient to warrant anticoagulation prophylaxis in most cases, suggesting that clinicians already try to target higher-risk anterior STEMI patients for LVT prophylaxis. However, while not powered for subgroup analyses, the findings in the different LV dysfunction subgroups were generally similar to the overall results in this analysis.

The results of this retrospective study must be interpreted with caution, however, and should be viewed as hypothesis generating; potentially forming the basis for a definitive trial. First, only a minority of patients had a repeat TTE by 4 months and the use of contrast media was at the echocardiographer’s discretion. This issue is common to recent retrospective analyses^8,21,22^. Second, although clinical follow-up at 4 months was available for most patients, the occurrence of specific clinical events was not prospectively assessed or adjudicated. The low rates of LVT/SE^4,23,26^ and major bleeding, for example, raise at least the specter of ascertainment bias. Similarly, the “treatment failure” outcome, which was defined *post hoc*, is subject to the same risk of ascertainment bias as other outcomes. However, in contrast to other recent analyses on this subject, the risk of misclassifying treatment assignment is low and in-hospital events were not excluded. Additionally, the decision to include lower-risk anterior STEMI patients may have favored a strategy of less aggressive antithrombotic therapy (i.e. ticagrelor-DAPT). Finally, the null findings of the propensity matched analysis must be interpreted with caution due to sample size attrition and the low rate of clinical events.

Despite these limitations, both the present dual-center analysis and the single-center analysis by Le May *et al*.^8^ failed to show an advantage for TATT over DAPT when prescribed solely for apical dysfunction following STEMI in a pPCI population. Shavadia *et al*.^22^ had similar findings in their propensity-matched analysis of a mixed pPCI/pharmacoinvasive population. Le May *et al*.’s study even suggested the possibility of increased harm with TATT when compared to clopidogrel-DAPT, with hemorrhagic events as the main driver^8^. Our analysis also showed an excess of bleeding with a selective TATT strategy. As the hemorrhagic risk of TATT compared to DAPT is well-documented^1,4,27,28^, this is perhaps not surprising. (A similar numerical tendency was noted by Shavadia *et al*. that was not statistically significant^22^). In contrast to the study by Le May *et al*.^8^, however, unplanned revascularization – and not bleeding –was the primary driver of the NACE composite outcome excess post-discharge in our cohort. It is unclear why this would be, but foregoing the demonstrated benefit of ticagrelor- over clopidogrel-based therapy is a possibility, as is antithrombotic therapy interruptions resulting from undocumented major or non-major bleeding events.

In addition to the newer antiplatelet agents, such as ticagrelor, novel non-vitamin K antagonist oral anticoagulants (NOACs) are now available to clinicians, have been studied in patients requiring PCI^29–31^, and may ultimately result in alternative practical solutions for apical dysfunction without thrombus, as well. The PIONEER AF-PCI trial, for example, showed a reduced risk of bleeding without an increased risk of ischemic events using either of two rivaroxaban regimens in atrial fibrillation patients requiring PCI compared to conventional TATT^32^, making these interesting strategies for study in high-risk anterior STEMI populations.

## Conclusion

The present study adds significantly to the literature on anticoagulation for apical dysfunction in the pPCI era, including predictors of hemorrhagic and ischemic events, and is the first to compare a strategy of systematic ticagrelor-DAPT to a traditional one of selective (physician discretion) TATT. When applied to a broad anterior STEMI population (≥1 dysfuncitonal apical segment regardless of LVEF), selective TATT was not shown to be superior to ticagrelor-DAPT and may be associated with excess harm. However, the occurrence of LVT in the ticagrelor-DAPT group deserves consideration and close echocardiographic follow-up of patients not treated with TATT may well be prudent. Given the methodological limitations of retrospective analyses published to date in pPCI populations and the expanded antithrombotic armamentarium currently available, an appropriately powered prospective trial is warranted.

## Electronic supplementary material

Supplementary Material
