# Supplementary material for: Comparison of Systematic Ticagrelor-Based Dual Antiplatelet Therapy to Selective Triple Antithrombotic Therapy for Left Ventricle Dysfunction Following Anterior STEMI
Source: Sci Rep. 2018 Jul 9;8:10326. doi: 10.1038/s41598-018-28676-4 (PMC6037676; doi:10.1038/s41598-018-28676-4)
Supplement: Supplementary file 1 — Supplementary Material [file 41598_2018_28676_MOESM1_ESM.docx]

**Appendix**

**(Supplementary Material)**

Comparison of Systematic Ticagrelor-Based Dual Antiplatelet Therapy to

Selective Triple Antithrombotic Therapy for Left Ventricle Dysfunction Following Anterior STEMI.

Alexandra Bastiany MD,^a^ Alexis Matteau MD SM,^a,b^ Fady El-Turaby MD,^a^

Alexandre Angers-Goulet MD,^c^ Samer Mansour MD,^a,b^ Benoit Daneault MD,^c^

& Brian J. Potter MDCM SM.^a,b^

1. Cardiology Service, Department of Medicine, Centre Hospitalier de l’Université de Montréal (CHUM), Montréal, QC, Canada.
2. Centre de Recherche du CHUM (CRCHUM), Montréal, QC, Canada.
3. Cardiology Service, Department of Medicine, Centre Hospitalier de l’Université de Sherbrooke (CHUS), Sherbrooke, QC, Canada.

**Author of correspondence:**

Brian J. Potter, MDCM SM FRCPC

Centre de recherche du CHUM (CRCHUM)

850, rue Saint-Denis

Pavillon S, S03-344

Montréal, QC, Canada

H2X 0A9

Tel: 514-890-8000 ext. 15471

Fax: 514-412-7212

brian.potter@umontreal.ca

**Table A1.** Selected Primary and Secondary Post-Discharge Outcomes in High Thrombotic Risk Sub-Cohorts of the Intention to Treat Population.

|  | CHUM  (Selective TATT) | CHUS  (Ticagrelor-DAPT) | Unadjusted  p-value |
| --- | --- | --- | --- |
| Overall Population | N=64* | N=108 |  |
| NACE | 17 (27%) | 21 (19%) | 0.342 |
| MACCE | 13 (21%) | 21 (19%) | 0.844 |
| Major Bleeding | 4 (7%) | 0 (0%) | 0.019 |
| Irreversible Events | 9 (15%) | 7 (6%) | 0.104 |
| LVT | 0 (0%) | 1 (2%) | 1.000 |
|  |  |  |  |
| ≥ 2 Apical Segments Only | N=58 | N=95 |  |
| NACE | 7 (12%) | 0 (0%) | <0.001 |
| MACCE | 7 (12%) | 0 (0%) | <0.001 |
| Major Bleeding | 0 (0%) | 0 (0%) | - |
| Irreversible Events | 4 (7%) | 0 (0%) | 0.019 |
| LVT | 0 (0%) | 0 (0%) | - |
|  |  |  |  |
| LVEF ≤40% Only | N=29 | N=70 |  |
| NACE | 3 (10%) | 0 (0%) | 0.023 |
| MACCE | 3 (10%) | 0 (0%) | 0.023 |
| Major Bleeding | 0 (0%) | 0 (0%) | - |
| Irreversible Events | 3 (10%) | 0 (0%) | 0.023 |
| LVT | 0 (0%) | 0 (0%) | - |
|  |  |  |  |
| LVEF ≤40% & ≥2 Segs. Only | N=29 | N=70 |  |
| NACE | 3 (10%) | 0 (0%) | 0.023 |
| MACCE | 3 (10%) | 0 (0%) | 0.023 |
| Major Bleeding | 0 (0%) | 0 (0%) | - |
| Irreversible Events | 3 (10%) | 0 (0%) | 0.023 |
| LVT | 0 (0%) | 0 (0%) | - |

**Table A2.** Selected Primary and Secondary Post-Discharge Outcomes in the Survivorship Sub-Cohort.

|  | CHUM  (Selective TATT) | CHUS  (Ticagrelor-DAPT) | Unadjusted  p-value |
| --- | --- | --- | --- |
| Overall Population | N=59* | N=101 |  |
| NACE | 7 (12%) | 0 (0%) | <0.001 |
| MACCE | 7 (12%) | 0 (0%) | <0.001 |
| Major Bleeding | 0 (0%) | 0 (0%) | - |
| Irreversible Events | 4 (7%) | 0 (0%) | 0.017 |
| LVT | 0 (0%) | 1 (2%) | 1.000 |
|  |  |  |  |
| ≥ 2 Apical Segments Only | N=58 | N=95 |  |
| NACE | 7 (12%) | 0 (0%) | <0.001 |
| MACCE | 7 (12%) | 0 (0%) | <0.001 |
| Major Bleeding | 0 (0%) | 0 (0%) | - |
| Irreversible Events | 4 (7%) | 0 (0%) | 0.019 |
| LVT | 0 (0%) | 0 (0%) | - |
|  |  |  |  |
| LVEF ≤40% Only | N=29 | N=70 |  |
| NACE | 3 (10%) | 0 (0%) | 0.023 |
| MACCE | 3 (10%) | 0 (0%) | 0.023 |
| Major Bleeding | 0 (0%) | 0 (0%) | - |
| Irreversible Events | 3 (10%) | 0 (0%) | 0.023 |
| LVT | 0 (0%) | 0 (0%) | - |
|  |  |  |  |
| LVEF ≤40% & ≥2 Segs. Only | N=29 | N=70 |  |
| NACE | 3 (10%) | 0 (0%) | 0.023 |
| MACCE | 3 (10%) | 0 (0%) | 0.023 |
| Major Bleeding | 0 (0%) | 0 (0%) | - |
| Irreversible Events | 3 (10%) | 0 (0%) | 0.023 |
| LVT | 0 (0%) | 0 (0%) | - |

*Seven survivorship sub-cohort patients at the CHUM patients did not have clinical follow-up within 4±1month post-MI at either the CHUM or the identified referring center.

NACE: Net adverse clinical events. MACCE: Major adverse cardiovascular and cerebrovascular events. LVT: Left ventricular thrombus. LVEF: Left ventricular ejection fraction.

**Propensity-Matched Analysis**

Using SAS 9.4 (SAS Institute Inc., Cary, North Carolina), a propensity score was derived for all patients for the likelihood of receiving anticoagulation at discharge, irrespective of the center of admission, based on clinical variables available to the treating physician at the time of discharge, including baseline characteristics, laboratory values, procedural details, TTE results, and in-hospital clinical events. Patients not surviving to discharge were not eligible for the propensity-matched analysis. Anticoagulated patients (from either center) were matched to non-anticoagulated patients (from either center) using the propensity scores and a greedy matching algorithm. The resulting propensity-matched cohort was comprised of 21 matched pairs. There were no significant differences in the pre-discharge variables apart from a larger proportion of TATT patients being derived from the CHUM population compared to the matched DAPT population (81% vs 38%, p=0.011) and a higher proportion of ticagrelor use among DAPT compared to TATT patients (62% vs 5%, p<0.001), as anticipated. All clinical composite outcomes at 4 months were both rare and not different between the groups. Selected baseline patient characteristics, procedural details, discharge antiplatelet therapy, and clinical events at 4 months are shown in Table A1.

**Table A3. Baseline and procedural characteristics and clinical outcomes in the propensity-matched cohort.**

|  | **TATT**  (N=21) | **DAPT**  (N=21) | **p-value** |
| --- | --- | --- | --- |
| Age | 62.3±11.3 | 61.8±14.1 | NS |
| Men | 14 (67%) | 15 (71%) | NS |
| Diabetes | 6 (29%) | 7 (33%) | NS |
| Hypertension | 11 (52%) | 10 (48%) | NS |
| Dyslipidemia | 8 (38%) | 10 (48%) | NS |
| BMI | 27.3±3.7 | 27.6±3.4 | NS |
| eGFR | 92.7±34.3 | 102.4±41.3 | NS |
| Hemoglobin | 140.4±15.2 | 144.2±18.6 | NS |
| CHA_2_DS_2_-VASC Score | 1 (0-2) | 1 (0-2) | NS |
| HASBLED Score | 1 (0-2) | 1 (0-2) | NS |
| CHUM Site | 17 (81%) | 8 (38%) | 0.011 |
| Drug-Eluting Stent | 17 (81%) | 15 (71%) | NS |
| P2Y12-Inhibitor  Clopidogrel  Prasugrel  Ticagrelor | 16 (76%)  3 (14%)  1 (5%) | 3 (14%)  4 (19%)  13 (62%) | <0.001  NS  <0.001 |
| 4-month Follow-up Available | 20 (95%) | 18 (86%) | NS |
| “Net Benefit” | 1 (5%) | 1 (6%) | NS |
| MACCE | 1 (5%) | 1 (6%) | NS |
| Major Bleeding | 0 (0%) | 0 (0%) | - |
| “Irreversible” | 1 (5%) | 0 (0%) | NS |
| “Treatment Failure” | 1 (5%) | 1 (6%) | NS |

TATT: Triple antithrombotic therapy. DAPT: Dual antiplatelet therapy. BMI: Body mass index. eGFR: Estimated glomerular filtration rate (Cockcroft-Gault method). CHUM: Centre hospitalier de l’Université de Montréal. PCI: Percutaneous coronary intervention. MACCE: Major Adverse Cardiovascular and Cerebrovascular Events.
